# Progressive Multifocal Leukoencephalopathy in a Patient With Classic Hodgkin’s Lymphoma Post-Bone Marrow Transplant: A Case Report

**DOI:** 10.7759/cureus.33473

**Published:** 2023-01-07

**Authors:** Ibad Rehman, Zafar Ali, Sana Rasheed, Irfan Ullah, Abdulqadir J Nashwan

**Affiliations:** 1 Student, Shifa International Hospital Islamabad, Islamabad, PAK; 2 Histopathology, Shifa International Hospital Islamabad, Islamabad, PAK; 3 Internal Medicine, Shifa International Hospital Islamabad, Islamabad, PAK; 4 Internal Medicine, Kabir Medical College, Peshawar, PAK; 5 Nursing Department, Hamad Medical Corporation, Doha, QAT

**Keywords:** non-hodgkin's lymphoma, hodgkin's lymphoma, bone marrow transplant, post bone marrow transplant complications, multifocal leukoencephalopathy

## Abstract

A 29-year-old male patient underwent an autologous bone marrow transplant. He was initially diagnosed with Hodgkin's lymphoma and treated with 12 cycles of chemotherapy. Three months later, he presented with intermittent fever and underwent an MRI scan and a brain biopsy. Eventually, he was diagnosed with progressive multifocal leukoencephalopathy. For effective treatment and a plan of action, such cases necessitate multidisciplinary board meetings with input from experts in surgery, pathology, cancer, and infectious diseases.

## Introduction

Progressive multifocal leukoencephalopathy (PML) is a severe demyelinating disorder of the nervous system that was first observed in a patient with chronic lymphocytic leukemia in 1958 [1,2]. Primary infection is mostly asymptomatic and occurs most commonly in childhood [2]. The John Cunningham virus (JCV) is transported to the kidney and lymphoid organs during primary infection and remains latent there. It is a very rare disease that mostly only affects people with weak immune systems, such as those who have had organ transplants or are receiving immunomodulatory therapy [2]. Its association with transplant recipients is significant since a study showed that even though bone marrow recipients have better chances of survival than solid organ recipients, there is no definite treatment with demonstrable therapeutic benefits [3,4]. Patients commonly present with neurological deficits such as diplopia, motor deficits such as hemiparesis, and even altered mental status [4]. The presentation may vary according to the stage and progress of the disease. After initial blood studies such as complete blood differential and imaging such as brain MRI, it is diagnosed by cerebrospinal fluid (CSF) analysis via lumbar puncture and polymerase chain reaction (PCR) for JCV [1-4].

The rationale for reporting this case lies in the fact that other than being an extreme entity, to the best of our knowledge, it is among the first few cases being reported in young males from Southeast Asia. This case report shows that more research needs to be conducted on progressive multifocal encephalopathies.

This article has been posted as a preprint on Authorea [5].

## Case presentation

The patient is a 29-year-old male, Asian, who presented with complaints of fever, weight loss, decreased appetite, and weakness. Initial workup revealed multiple liver and spleen lesions on abdominal ultrasound. Further investigations that included a liver biopsy proved to be inconclusive. The patient was referred to our hospital for a bone marrow biopsy, which showed classic Hodgkin's lymphoma features. He received his complete six cycles of chemotherapy: Adriamycin, Bleomycin sulfate, Vinblastine sulfate, and Dacarbazine (ABVD). This was followed by a repeat positron emission tomography/computed tomography (PET/CT) scan. The PET/CT post-ABVD showed progressive disease above and below the diaphragm, including sub-aortic nodes, para-aortic nodular involvement, and osseous marrow involvement (Deauville 5). A second vertebral biopsy was performed and found to be CD20 and CD30 positive. After reassessment, he was given a further 4 cycles of rituximab, ifosfamide, carboplatin, and etoposide (R-ICE). A post-therapy PET scan was performed and was read as a complete metabolic response (Deauville 3). He was advised to have an emergency transplant but failed to follow through.

After six months, he presented with severe anemia and dyspnea. Consequently, the patient was transfused with blood and improved symptomatically. PET/CT was performed, which showed progressive disease (Deauville 5) with interval development of below diaphragm nodal disease in the inguinal left iliac, aortic, celiac, superior mesenteric nodes, and heterogonous marrow uptake. He was started on rituximab-bendamustine (R-Benda) and completed three cycles within the next three months. Ct/PET after R-Benda showed a complete metabolic response (Deauville 2).

The patient was finally admitted to our hospital for an autologous bone marrow transplant planned for the same month. The BEAM conditioning protocol was delivered for six days. The patient tolerated them well. Patient harvesting was started and performed for three days, and a total of 2.42 million CD34 cells were extracted with cell viability of 86%.

Stem cells were transfused in the same week, and engraftment was initiated. Post-engraftment, the patient remained stable and was subsequently discharged home with guidance regarding transfusions for low hemoglobin and neutropenic fever. On follow-up visits for the next three months, the patient remained stable.

**Figure 1:**
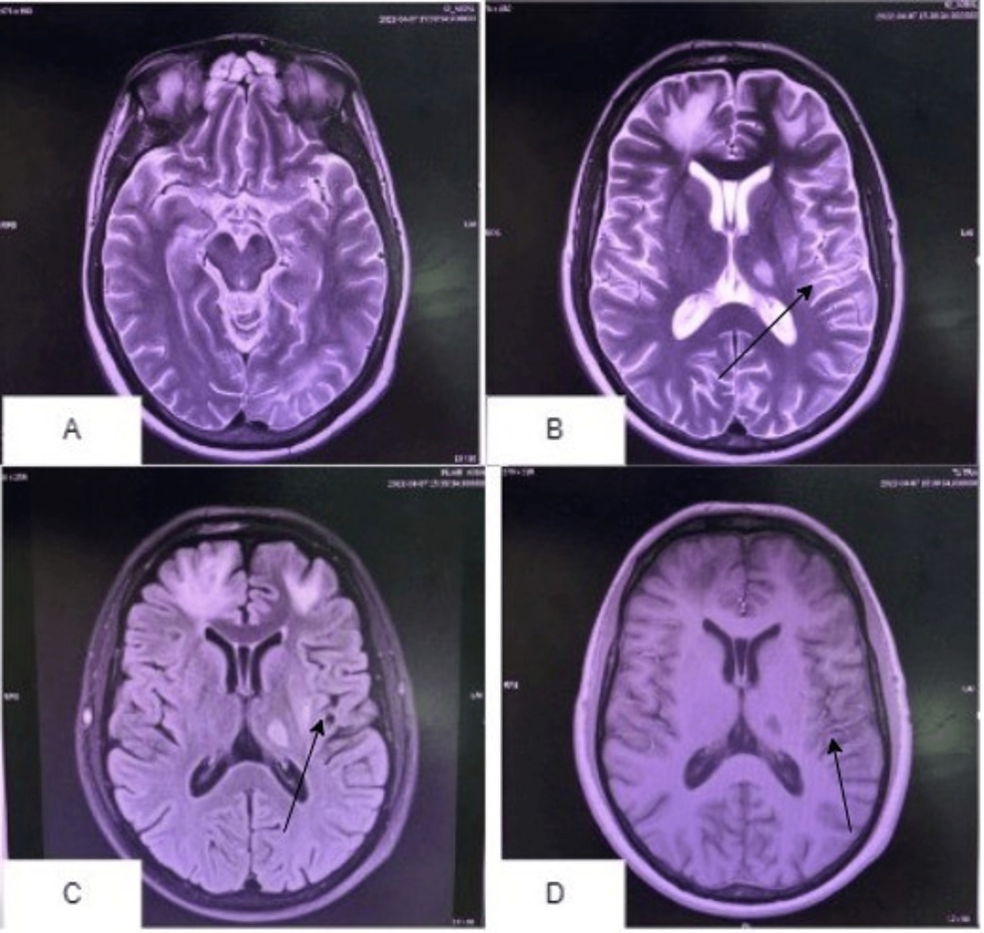
Brain multi-sequential MRI Multiplanar, multi-sequential MRI of the brain with and without contrast in images A, B, C, and D showing interval development of small T2 and FLAIR hyperintense signals noted in the left peri-insular region (C and D) and in the left medial temporal lobe (B).

He presented after three months with a complaint of intermittent fever and forgetfulness for one week. His CT showed T2 and FLAIR hyperintense signal lesions in the bilateral periventricular and subcortical white matter of the frontal lobes, left frontoparietal lobe, left occipital lobe, and left thalamus (Figure 1); findings were consistent with PML with disease progression, and right frontal craniotomy was performed for biopsy.

**Figure 2:**
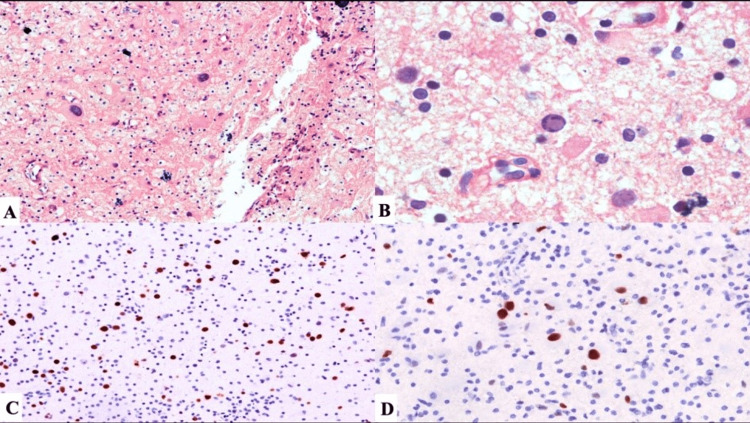
Progressive multifocal leukoencephalopathy (PML) (A) Bizarre, pleomorphic multilobulated astrocytic nuclei in the background of histiocytes is a characteristic feature of PML (H&E, 20×). (B) Inclusion-bearing, enlarged nuclei in virally infected oligodendrocytes with a peripheral displacement of native nuclear chromatin (H&E, 40×). (C) SV40 viral protein immunohistochemistry demonstrates immunoreactivity in enlarged and small oligodendroglial nuclei (H&E, 20×). (D) Virally infected cells bind p53 and demonstrate nuclear immunoreactivity (H&E, 40×).

The biopsy showed bizarre, pleomorphic, multilobulated astrocytic nuclei in the background of histiocytes, which is a characteristic feature of PML (Figure 2).

The post-operative patient remained stable and was discharged with advice regarding wound care and home medications, which included a tapering dose of dexamethasone. The patient was also advised to follow up in the outpatient department after one week, for which he did not show up.

## Discussion

PML is a neuroinfectious disease caused by the JCV, which belongs to the papovavirus family [1]. One of the major reasons for a rise in PML patients is the human immunodeficiency virus (HIV) and the infection it causes, although antiretroviral therapy proved to be very beneficial in managing such patients. There are other conditions that are often associated with the activation of the JCV, but a major chunk of cases have been associated with HIV infection. A rise in this condition has been observed with advances in immunomodulatory therapies. One such example is natalizumab, which has a dual inhibition role, hence making it a probable reason for developing PML [1-4].

PML should be suspected in cases of rapidly progressing neurological symptoms and deterioration in immunocompromised individuals. Transplant recipients are at a small but significant risk of developing PML. They have the greatest risk of disease onset immediately post-transplantation. This risk decreases smoothly thereafter and eventually stabilizes and persists lifelong [4,6]. In our case, the patient developed neurological symptoms three months after the autologous bone marrow transplant. Regardless, PML must be suspected at all times during the post-transplantation period [7].

In our patient, cognitive decline, particularly amnesia, was the predominant symptom, along with fever. Additionally, typical radiological findings on brain CT or MRI support the diagnosis. In the present case, a CT of the brain was performed, which showed T2 and FLAIR hyperintense signal lesions in the bilateral periventricular and subcortical white matter of the frontal lobes, left frontoparietal lobe, left occipital lobe, and left thalamus. The findings suggest PML as the disease progresses. Clinically, diagnosing PML can be a very perplexing task because depending on the part of the brain being affected, its presentation can vary; the majority of cases, though, show a mix of decreases in cognition, visual disabilities, and speech disorders [8-10].

The rationale for suspecting PML in our patient was based on clinical and imaging findings in the background of an immunocompromised state that prompted us to proceed with the brain biopsy. It is reported as bizarre, pleomorphic, multilobulated astrocytic nuclei in the background of histiocytes, which is a characteristic feature of PML. These features on biopsy are very characteristic of JCV infection, as reported previously [9-11].

Sometimes, the underlying diagnosis prompting transplantation, including Hodgkin's lymphoma and other conditions, has been associated with the development of PML even in the absence of transplantation and immunosuppressive drugs [12]. Although PML can occur in hematological malignancies even in the absence of transplantation, as reported by Ström et al. [4], transplantation and immunosuppression, rather than the underlying disease, are the most important determinants for the development of PML [10-12]. Regardless, a history of pre-existing disease such as lymphoma causes diagnostic confusion regarding the recurrence of primary disease versus PML, a rare condition. Regardless, a high clinical suspicion and low diagnostic threshold for PML are keys to early diagnosis [13,14].

## Conclusions

Timely diagnosis of PML is important because the only way to stop the rapid decline of the patient's neurological status and improve survival rates is to try to restore the patient's immune status as soon as possible.
